# Editorial: COVID-19 Vaccines Safety Tracking (CoVaST): Part I

**DOI:** 10.3389/fpubh.2023.1154500

**Published:** 2023-02-24

**Authors:** Miloslav Klugar, Sameh Attia, Giordano Pérez-Gaxiola, Tina Poklepović Peričić, Janja Marc, Abanoub Riad

**Affiliations:** ^1^Czech National Centre for Evidence-Based Healthcare and Knowledge Translation (Cochrane Czech Republic, Czech EBHC: JBI Center of Excellence, Masaryk University GRADE Centre), Faculty of Medicine, Institute of Biostatistics and Analyses, Masaryk University, Brno, Czechia; ^2^Institute of Health Information and Statistics of the Czech Republic, Prague, Czechia; ^3^Department of Oral and Maxilofacial Surgery, Justus-Liebig University of Giessen, Giessen, Germany; ^4^Department of Evidence Based Medicine, Sinaloa Pediatric Hospital, Cochrane Mexico, Culiacán, Mexico; ^5^Department of Prosthodontics, Study of Dental Medicine, University of Split School of Medicine, Split, Croatia; ^6^Faculty of Pharmacy, University of Ljubljana, Ljubljana, Slovenia; ^7^Institute of Clinical Chemistry and Biochemistry, University Medical Centre Ljubljana, Ljubljana, Slovenia

**Keywords:** COVID-19, side effects, vaccination, cross-sectional study, VAERS, Vaccine Adverse Event Reporting System, systematic review

Acceleration of coronavirus disease (COVID-19) mass vaccination has been a chief priority for health systems globally since the first emergency approvals of COVID-19 vaccines in late 2020. Nevertheless, vaccine hesitancy (VH), which is nourished by misinformation about vaccines' effectiveness and safety, remains as a serious threat for vaccination strategies worldwide. Aversion to post-vaccination side effects, the lack of trust of pharmaceutical industry, and the lack of knowledge about vaccines' safety are among the VH drivers; therefore independent (non-sponsored) and active surveillance of COVID-19 vaccines safety is of utmost importance for suppressing VH.

This was a motivation for our team to initiate a global study which will be focused on the COVID-19 Vaccines Safety Tracking (CoVaST). We registered this study as the first of its kind with the US National Library of Medicine registry (ClinicalTrials.gov, accessed on 9 May 2021), with the identifier NCT04834869 and published the “*Protocol of a Multi-Center Prospective Cohort Study for Active Surveillance of COVID-19 Vaccines' Side Effects*” (1) together with our international partners from 24 institutions worldwide.

Our next logical step was the registration of the Research Topic with a prestigious, highly impactful journal focused on public health. The overarching aim of this Research Topic was to synthesize a collection of studies that evaluate the short-term side effects of different types of COVID-19 vaccines; i.e., mRNA-based, viral vector-based, inactivated virus-based, and protein subunit-based vaccines in various countries worldwide. The post-vaccination side effects can be evaluated either using the data of passive surveillance systems; e.g., VAERS, DAEN, EudraVigilance, etc., or through active surveillance (epidemiological) studies; e.g., cohort, cross-sectional studies, etc., and we were open also to research synthesis study designs.

We received 22 relevant submissions and accepted 15 articles after rigorous peer review and editorial process. Most of the accepted articles are epidemiological “active surveillance” studies, although three passive surveillance studies were included as well, together with two systematic reviews and two literature reviews (one with case series).

All included studies reported the good safety of all included vaccines against COVID-19. The prevalence of local and systemic side effects was modest. Most of the symptoms disappeared after 3 days. The risk-benefit ratio of vaccination remains positive compared to potential SARS-CoV-2 infection. Although all included studies except one systematic review, which meta-analyzed six randomized controlled trials with 6,427 participants in the observation group and 3,535 participants in the control group that reported safety data, are limited by their descriptive observational nature.

The mentioned systematic review from Du et al., evaluated the safety, immunogenicity, and efficacy of COVID-19 vaccines in adolescents, children and infants (0–17 years). Compared with mRNA vaccines and adenovirus vector vaccines, inactivated vaccines have a more satisfactory safety profile, both after the initial (RR 1.40, 95% CI 1.04–1.90) and booster (RR 1.84, 95% CI 1.20–2.81) vaccination. The risk of adverse events statistically significantly increased after the first and second doses, but there was no statistically significant difference between the first two doses (RR 1.00, 95% CI 0.99–1.02). Nevertheless, the two-dose regimen is obviously superior to the single-dose schedule for immunogenicity and efficacy. After booster vaccination, both neutralizing antibodies (RR 144.80, 95% CI 44.97–466.24) and RBD-binding antibodies (RR 101.50, 95% CI 6.44–1,600.76) reached optimal levels, but the cellular immune response did not appear to be further enhanced.

All descriptive cross-sectional studies of self-reported side effects consistently report local and systemic side effects with nuances coming from different types of vaccines, different age, and population groups. From Mexico Moll et al., (*n* = 4,024) at dose 1, ChAdOx1 was the vaccine with the highest rate of at least one side effect (85%) followed by Gam-COVID-Vac (80%). Both were associated with greater extension (adjusted OR 2.53, 95% CI 2.16, 2.96 and adjusted OR 2.41, 95% CI 1.76, 3.29, respectively) and severity of side effects (adjusted OR 4.32, 95% CI 3.73, 5.00, and adjusted OR 3.00, 95% CI 2.28, 3.94, respectively). Young age (< 50 years), female sex, comorbidity, and history of allergies were associated with greater extension and severity, independent of the type of vaccine and potential confounders. From 721 Algerian healthcare workers, Lounis et al., self-reported post-vaccination side effects of inactivated (BBIBP-CorV and CoronaVac) and adenoviral vector-based (AZD1222, Gam-COVID-Vac, and Ad26.COV2.S) vaccines. Less than half (49.1%) of the respondents reported at least one local side effect, while 53.8% reported at least one systemic side effect. These side effects were more prevalent among viral vector vaccinees than inactivated virus vaccinees. The side effects appeared earlier among inactivated virus vaccines recipients and generally lasted for 2–3 days for the two vaccinated groups. The risk factors associated with a higher prevalence of side effects included female gender, allergic individuals and individuals with regular medication. Data from Saudi Arabia on 1058 participants from the general population Al-Hanawi et al. observed that the most common vaccine side effects reported were tiredness/fatigue (52.6%), swelling (38%), fever (31.3%), headache (29.1%), and muscle pain (22.2%). In multivariable analyses, the odds of experiencing severe side effects were significantly higher among males [adjusted odds ratio (aOR) = 2.76, 95% confidence interval (CI) = 1.71–4.45, *p* < 0.01], those aged 40–49 years (aOR = 3.10, 95% CI = 1.10–8.72, *p* < 0.1), and Saudi nationals (aOR = 3.64, 95% CI = 1.58–8.38, *p* < 0.05) compared to their counterparts. Among those who had received two doses, a higher proportion had received Pfizer-BioNTech (54.2%) than AstraZeneca/Oxford (33.1%). Data from Ethiopia on a sample of 346 healthcare workers Yesuf et al., reported after the Oxford AstraZeneca COVID-19 vaccine prevalence of at least one local- and systemic-side effect was 50.6 and 44.5%, respectively. The most frequent local- and systemic- side effects were injection site pain and headache, respectively. Both types of side effects mostly subsided in the first 3 days. These data are consistent with other studies which used similar standardized tools as Yesuf et al. and Lounis et al. (2–6).

Another observational study among a population with stroke risk was reported on 1,747 participants from China Wu et al. the incidence of adverse events after the first and second dose was 16.6 and 13.7%, respectively. There was no difference in the incidence of adverse reactions among different risk groups. Sex, vaccine type, sleep quality, worry of adverse events, age, and education level were statistically significantly related to adverse reactions to vaccination.

One small observational study from Italy Reschini et al. on a sample of 106 men tested a hypothesis rather popular amongst conspirative theories regarding the impact of immunization on future fertility. The study concluded that no difference was observed even after considering different types of vaccines (viral vector or mRNA). The vaccination did not affect sperm quality and fertilization capacity of men undergoing assisted reproduction technology attempts.

Wound healing and scar formation were reported on small case series *n* = 31 to be not affected by the COVID-19 vaccination Dong et al. Case reports of four patients with myocarditis early after mRNA vaccination demonstrated the need for multimodal diagnostics, Nunn et al., however with certain limitations, authors concluded the risk-benefit ration of vaccination remains positive.

Three of included studies in our Research Topic were reporting the passive surveillance data based on the analyses of the Vaccine Adverse Event Reporting System (VAERS) co-managed by the United States Food and Drug Administration and the Centers for Diseases Control and Prevention. Analyses from Zou et al. showed that the most commonly reported adverse events of COVID-19 vaccines were mild. Cases with mortality outcomes tended to occur in older adults. However, the World Health Organization international database study did not identify significant safety concerns regarding mRNA vaccination in real-world settings. The authors reported an overall lower risk of serious adverse events following mRNA vaccines when compared to influenza vaccines. There were 103 (0.5%) deaths out of 18,755 COVID-19 vaccine-related AEs and 104 (0.4%) deaths out of 27,895 influenza vaccine-related AE (7). Bian et al. analyzed VAERS data from the perspective of allergic reactions after the COVID-19 vaccination and concluded that female predominance in allergic reaction cases after the receipt of COVID-19 vaccines was observed. Previous histories of allergies, asthma, or anaphylaxis were risk factors for anaphylaxis post-vaccination. Riad et al., focused their VAERS analyses on the oral adverse events following COVID-19 vaccination and reported that COVID-19 vaccines were found to be associated with rare oral adverse events that are predominantly similar to those emerging following seasonal influenza vaccines.

All included studies brought the best available evidence about short-term vaccine safety, which seems not to differ significantly from influenza vaccines. More evidence of the safety of COVID-19 vaccines is still needed to help make informed public decisions about their benefits, as there are newly developed booster doses of vaccines, and the virus is still mutating. It is important and challenging to collect longitudinal data about the safety of the COVID-19 vaccines.

## Author contributions

MK: drafted the manuscript. SA, GP-G, TP, JM, and AR: reviewed, edited, and approved the final manuscript. All authors contributed to the article and approved the submitted version.
